# The influence of aminophylline on the nanostructure and nanomechanics of T lymphocytes: an AFM study

**DOI:** 10.1186/1556-276X-9-518

**Published:** 2014-09-21

**Authors:** Xun Huang, Jiexiang He, Mingxian Liu, Changren Zhou

**Affiliations:** 1 Department of Materials Science and Engineering, Jinan University, Guangzhou 510630, China; 2 Engineering Research Center of Artificial Organs and Materials, Ministry of Education, Guangzhou 510630, China

**Keywords:** Atomic force microscopy, T lymphocytes, Aminophylline, Nanostructure, Nanomechanics

## Abstract

Although much progress has been made in the illustration of the mechanism of aminophylline (AM) treating asthma, there is no data about its effect on the nanostructure and nanomechanics of T lymphocytes. Here, we presented atomic force spectroscopy (AFM)-based investigations at the nanoscale level to address the above fundamental biophysical questions. As increasing AM treatment time, T lymphocytes' volume nearly double increased and then decreased. The changes of nanostructural features of the cell membrane, i.e., mean height of particles, root-mean-square roughness (Rq), crack and fragment appearance, increased with AM treatment time. T lymphocytes were completely destroyed with 96-h treatment, and they existed in the form of small fragments. Analysis of force-distance curves showed that the adhesion force of cell surface decreased significantly with the increase of AM treatment time, while the cell stiffness increased firstly and then decreased. These changes were closely correlated to the characteristics and process of cell oncosis. In total, these quantitative and qualitative changes of T lymphocytes' structure and nanomechanical properties suggested that AM could induce T lymphocyte oncosis to exert anti-inflammatory effects for treating asthma. These findings provide new insights into the T lymphocyte oncosis and the anti-inflammatory mechanism and immune regulation actions of AM.

## Background

Aminophylline (AM) is a complex of the bronchodilator theophylline [1]. AM has been used for treating asthma over 70 years [2,3]. Today, AM is still the main therapeutic agent in the treatment of asthma. Aminophylline has multiple potential beneficial actions in asthma, e.g., bronchial muscle relaxation, improvement in diaphragm contractility, diuretic function, and so on [4]. In addition, AM is a nonselective adenosine receptor antagonist and a phosphodiesterase (PDE) inhibitor that elevates intracellular levels of cyclic AMP (cAMP) [1]. High intracellular levels of cAMP can lead to the expansion of the smooth muscle [5]. So, the past view of AM in the treatment of bronchial asthma is mainly working as smooth muscle relaxant. In recent years, studies have shown that AM had widely anti-inflammatory and immune regulation actions [6-8], which may be the most important mechanism for the treatment of asthma.

T lymphocyte is one of the key players in the adaptive immune system and is involved in many kinds of inflammation [9,10]. Asthma is a chronic inflammatory disorder characterized by airway obstruction and hyper-responsiveness. The cells involved in the inflammatory response in asthma include lymphocytes, mast cells, eosinophils, macrophages, neutrophils, and epithelial cells [11]. So, the removal of the inflammatory cells is the key to radically cure asthma. The excessive activation of T cells is an important characteristic of asthma, because the activated T cells can release a variety of cytokines to activate B lymphocytes, macrophages, and so on [12]. So, the removal of T lymphocyte in the airway inflammation plays a key role in curing asthma. Most studies have suggested that apoptosis and oncosis are two crucial mechanisms for regulating immune and inflammatory responses [13]. The body massively weeds out the excess cells through oncosis [14]. In the past, AM treatments which induced the death mode of T lymphocyte have been studied biologically and/or biochemically. However, the quantitative analysis of morphological and nanostructural changes and the variations of cellular biomechanical properties in the context of AM treatment remain unclear yet because of the lack of sensitive quantitative techniques. In recent years, the nanostructure and mechanical properties of cells are becoming as indicators and regulators of physiological processes such as activation, differentiation, apoptosis, malignant phenotypes, and mitosis [10,15-17]. Therefore, measurements of the morphology, the nanostructure, and the biomechanical properties of T lymphocyte treated with AM for different times at the nanoscale level are of major importance to further interpret the death mode of T lymphocyte in asthma and may lead to new insightful views to the mechanism of AM treating asthma.

Atomic force microscopy (AFM) is a powerful nanotechnology tool that has been applied to observe nanostructural details and biomechanical properties of biological samples including biomolecules, cells, and biomaterials [18-22] and has been demonstrated as a useful nanotechnology for obtaining dynamic processes of ligand/receptor and cell-cell interaction [18,22,23]. The ultra-high force sensitivity of AFM and its ability to measure nanomechanical properties of individual cell make the technique particularly appropriate for measuring the changes of viscoelasticity including the adhesion force, elasticity, and stiffness of cells [24,25]. The studies on biophysical properties (morphology, nanostructure, adhesion force, stiffness, and others) of cells' near physiological conditions will provide fundamental insights into cellular structures and biology functions. In this study, AFM and AFM-based force spectroscopy were applied to characterize the morphology and the nanostructure and to measure the adhesion force and stiffness of T lymphocyte treatment with AM for different times *in vitro*. Obviously, this study would provide a novel insight into the mechanism of AM treatment of asthma at the single-cell level and the nanoscale level.

## Methods

### Preparation of T lymphocyte

BALB/c mice (6 to 8 weeks old) were used for the experiments. The abdomen was opened and the spleen was removed under sterile conditions. Mononuclear cells were prepared following mechanical spleen smash on a stainless steel mesh using complete RPMI-1640 culture medium (containing 10% fetal bovine serum, 2 mM l-glutamine, 25 mg/ml penicillin G, and 25 mg/ml streptomycin). T lymphocyte was isolated from mononuclear cells by nylon fiber column. Cell suspensions were adjusted to a final concentration of 1.4 × 10^6^ cells/ml. Cells were cultured in RPMI-1640 culture medium in a humidified incubator (37°C, 5% CO_2_).

### Aminophylline treatment and culture of T lymphocyte

Aminophylline treatment T lymphocyte experiments were performed in a 24-well tissue culture plate. Each well of the plate contained about 1.4 × 10^6^ cells in 200 ml RPMI-FBS medium. Cells were incubated with 10 μg/ml aminophylline and cultured at 37°C (5% CO_2_) in a humidified incubator for 48, 72, and 96 h, respectively.

### Sample preparation for AFM

To detect the morphological and mechanical changes of T lymphocyte before and after AM treatment, cells were separated into four groups: AM-free culture medium group and 10 μg/ml AM treatment for 48, 72, and 96 h groups. They were then washed in distilled water twice before being fixed with 4% paraformaldehyde for 20 min. These samples were washed in distilled water three times again and then air-dried for AFM scanning.

### AFM measurements

All the morphology images and force-distance curves were obtained by AFM (Autoprobe CP Research, Thermomicroscopes, Sunnyvale, CA, USA) in contact mode. All force-distance curve experiments were performed at the same loading rate. The curvature radius of the silicon tip is less than 10 nm, the force constant is 0.6 N/m, and the scan rate is 0.3 to 1 Hz. Over 1,000 force curves were acquired for each sample, and all the force curves were performed with the same tip. About morphology images, more than 20 cells were investigated with AFM for each sample.

### Data processing and statistics

The acquired images (256 × 256 pixels) were only processed with the instrument-equipped software (Thermo-microscopes Proscan Image Processing Software Version 2.1, IP 2.1) to eliminate low-frequency background noise (flatten order: 0 to 2) in the scanning direction. The quantitative analysis of root-mean-square roughness (Rq), cell volume, cell height, full width at half maximum (FWHM), and mean height of particles was also acquired by the above AFM software. The data were reported as mean ± SD, and data analysis was conducted using Origin 8.5 software. Differences with *P* < 0.05 were regarded statistically significant. The stiffness of cell membrane can be qualitatively evaluated based on the slope of compliance portion of the force-distance curve [26,27].

## Results and discussion

### Morphology and nanostructure of T lymphocytes

**Figure 1:**
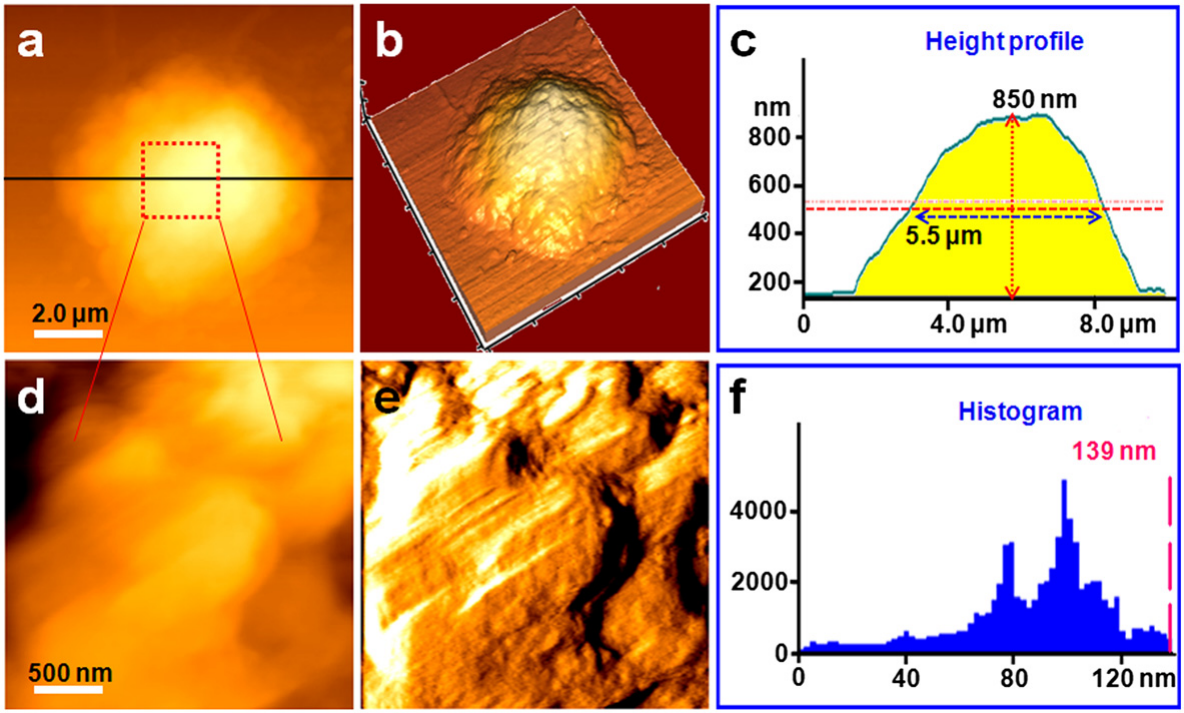
**Representative AFM images of normal T lymphocytes. (a)** Topological morphology image of a normal T lymphocyte, **(b)** 3-D image of **(a)**, and **(c)** height profile of **(a)**. **(d)** Nanostructure of the cell membrane surface zoomed from **(a)**, **(e)** error signal mode image of **(d)**, and **(f)** histogram of the particles' diameter of **(d)**.

**Figure 2:**
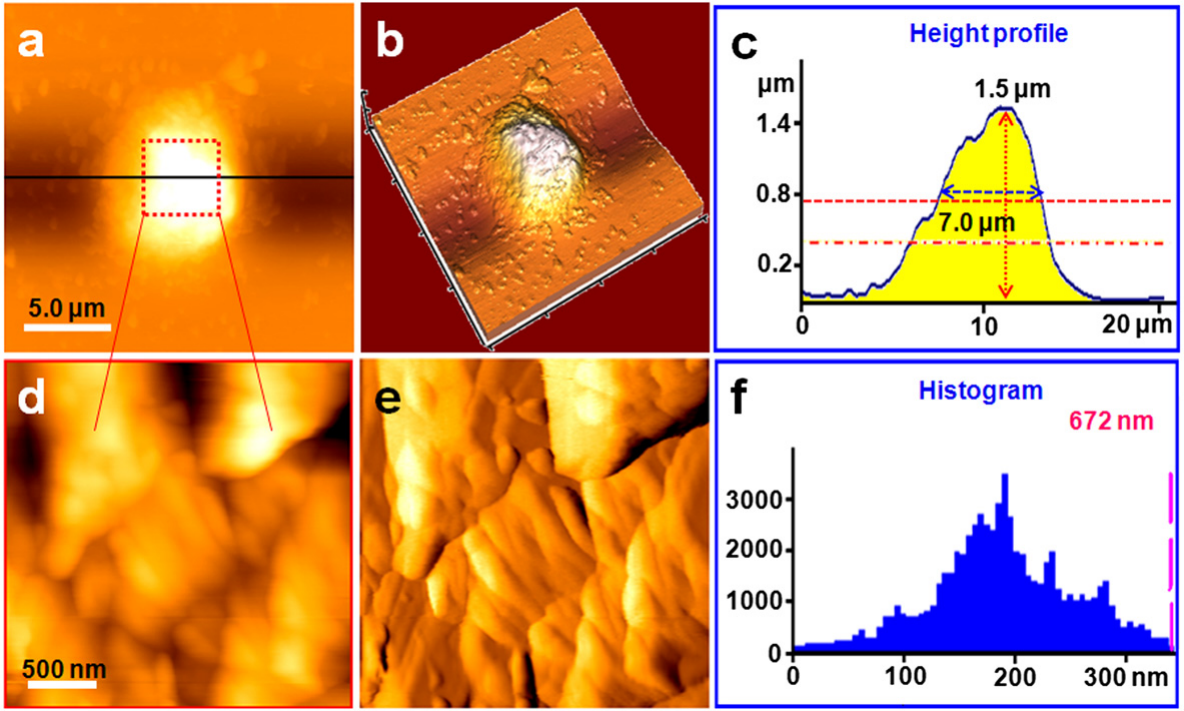
**Representative AFM images of T lymphocytes treated with AM for 48 h. (a)** Topological morphology image of a T lymphocyte treated with AM for 48 h, **(b)** 3-D image of **(a)**, and **(c)** height profile of **(a). (d)** Nanostructure of the cell membrane surface zoomed from **(a)**, **(e)** error signal mode image of **(d)**, and **(f)** histogram of the particles' diameter of **(d)**.

**Figure 3:**
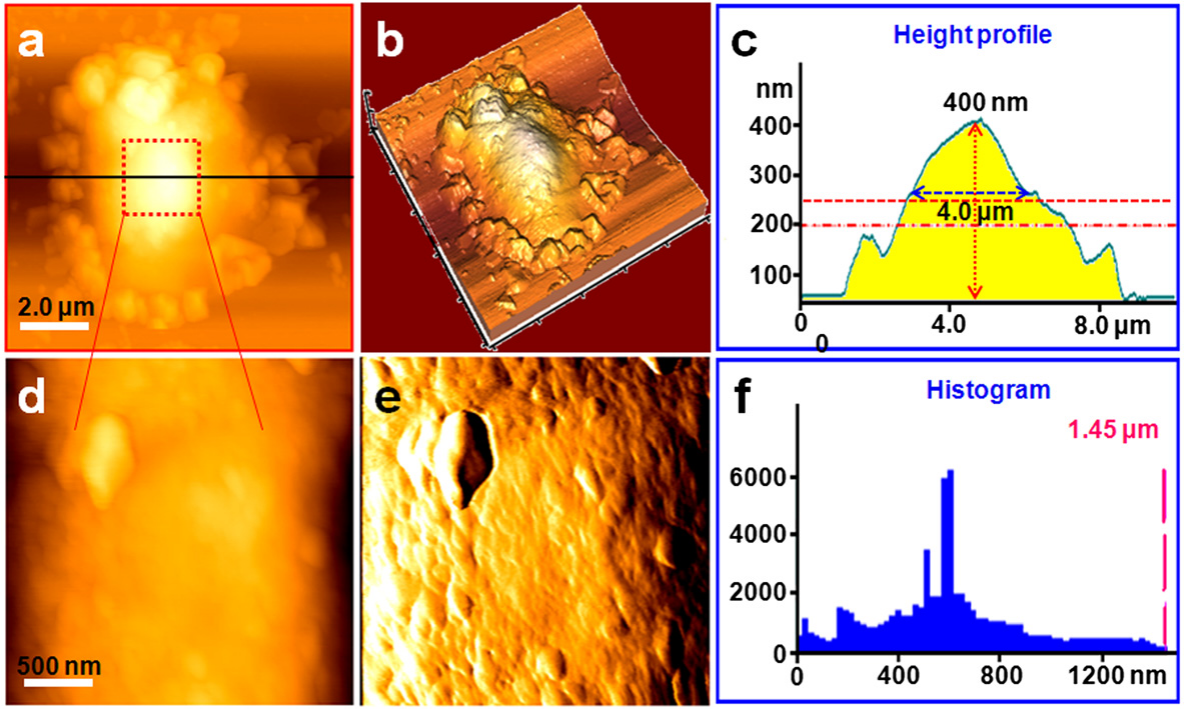
**Representative AFM images of T lymphocytes treated with AM for 72 h. (a)** Topological morphology image of a T lymphocyte treated with AM for 72 h, **(b)** 3-D image of **(a)**, and **(c)** height profile of **(a)**. **(d)** Nanostructure of the cell membrane surface zoomed from **(a)**, **(e)** error signal mode image of **(d)**, and **(f)** histogram of the particles' diameter of **(d)**.

**Figure 4:**
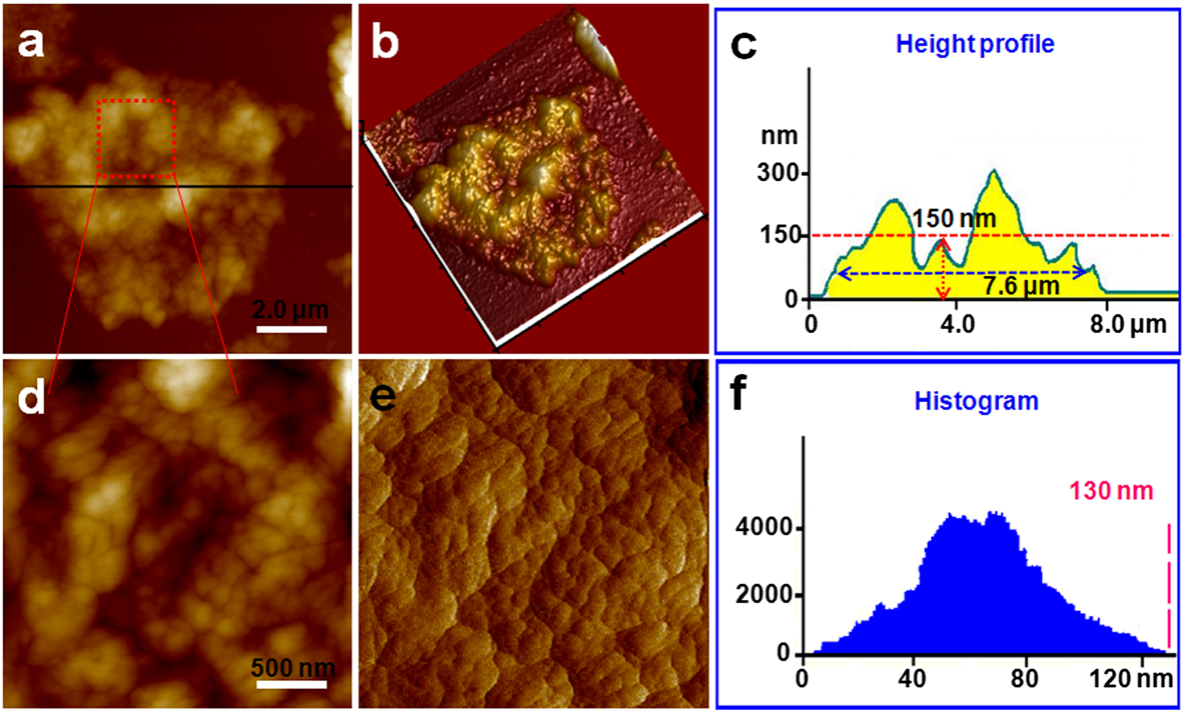
**Representative AFM images of T lymphocytes treated with AM for 96 h. (a)** Topological image of a T lymphocyte treated with AM for 96 h, **(b)** 3-D image of **(a)**, and **(c)** height profile of **(a)**. **(d)** Nanostructure of the cell membrane surface zoomed from **(a)**, **(e)** error signal mode image of **(d)**, and **(f)** histogram of the particles' diameter of **(d)**.

Each cell of an organism has its specific size and shape which plays a specific function. When the cells' morphology and nanostructure changed, the cells would play another function, e.g., resting and activated T cells [10] and immature and mature dendritic cells [28]. Integrity of the cell membrane is a basic requirement for maintaining the biological characteristics and physiological function of cells [29]. Disruptions in cell membrane structure can therefore directly influence the normal functions of cells [30]. In *in vitro* studies, changes in the general morphology of cells under the effects of medicaments are commonly used as a basis for judgment of drug effects and to identify the way of cell death [31]. AFM has unparalleled resolution compared with the traditional optical microscope, which can not only detect cell morphology but also explore real-time changes on the nanostructure of cells.With this in mind, changes in the morphology and nanostructure of T lymphocyte were first studied by AFM both before (Figure 1) and after treatment with AM for 48 h (Figure 2), 72 h (Figure 3), and 96 h (Figure 4). Normal T lymphocytes presented regular spherical shape (Figure 1a,b). Figure 1a shows a topography image which displayed the height of T lymphocytes by the change of color from dark to light. Figure 1b shows a three-dimensional (3-D) image of Figure 1a, which intuitively showed the cell shape. Importantly, in the 3-D image, the structural details like pseudopodia and cellular microvilli could be more easily distinguished (Figure 1b). Figure 1c presents a height profile generated along the black line in Figure 1a, and the measurement indicated the cell size with a height of 850 nm and FWHM of 5.5 μm. FWHM could be used to indicate the diameter of cells. Figure 1d,e vividly revealed the nanostructure of the cell membrane, indicating the relatively smooth and intact membrane surface structure. Figure 1e shows an error signal mode image, which showed the wavy structure of normal T lymphocytes. This special structure may be caused by the existence of microvilli, which further proved the intactness of cell membrane nanostructure. The cell membrane nanostructure components were uniformly distributed and granular. The particles in the cell membrane surface are mainly composed of carbohydrates and proteins. The histogram showed that the particles' diameter mainly ranged from 40 to 120 nm, but most of the particles are 100 nm in diameter (Figure 1f).

### Changes in morphology and nanostructure of T lymphocytes following AM treatment

As seen in Figures 2, 3, and 4, following treatment with 10 μg/ml AM, the T lymphocyte surface morphology and nanostructure began to change obviously with increasing treatment time. Figure 2a,b,c,d,e,f shows the morphology and nanostructure of T lymphocytes exposed to 10 μg/ml AM for 48 h. Compared with normal T lymphocytes, treatment with 10 μg/ml AM for 48 h resulted in membrane disruption, and the cells' shape became irregular and the membrane surface was uneven (Figure 2a,b). The pseudopodia and cellular microvilli all disappeared, and some debris (500 nm to 2 μm in diameter) was found on the cell surface and the substrate (Figure 2b). As expected, the cells became significantly larger in height (1.5 μm) and FWHM (7.0 μm) after being treated with AM for 48 h (Figure 2c). Simultaneously, the nanostructure of the cell membrane surface was changed (Figure 2d,e) compared with normal T lymphocytes (Figure 1d,e). Several cracks and pores were also found in the cell membrane surface (Figure 2d). In addition, the particles in the cell membrane became larger and more heterogeneous (Figure 2e). As shown in Figure 2f, the particles' diameter mainly ranged from 100 to 300 nm, and the largest particle was over 672 nm in diameter.As shown in Figure 3, when the treatment time was increased to 72 h, T lymphocytes were severely damaged (Figure 3a,b). The cells' shape became more irregular, and there were some larger and thicker fragments around the cells compared with T lymphocytes that were exposed to 10 μg/ml AM for 48 h (Figure 2a,b). By contrast, the cells' height and FWHM both decreased obviously (Figure 3c). The surface nanostructure (Figure 3d,e) was completely different from that of normal T lymphocytes (Figure 1d,e), and a few big clusters appeared on the cell surface (Figure 3e). The particles in the surface became more unevenly distributed with the diameter ranging from 100 to 1,200 nm, and the largest particle cluster was over 1.45 μm in diameter (Figure 3f).In order to test whether T lymphocytes were completely lysed with increasing treatment time, we further detected the morphology and nanostructure of T lymphocytes treated with AM for 96 h (Figure 4). As expected, AFM images showed that the cells were completely destroyed, and they all existed in the form of fragments (Figure 4a,b). The cell height decreased apparently with the height only about 150 nm (Figure 4c). Fine structure showed that the cell fragments became smaller (Figure 4d,e,f). These visual data demonstrated that T lymphocytes were completely lysed when exposed to 10 μg/ml AM treatment for 96 h.

### Quantitative analysis of the biophysical parameters of T lymphocytes

**Figure 5:**
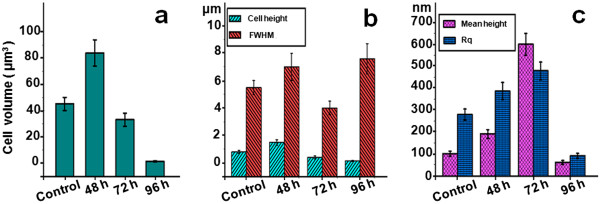
**Histograms of statistical results about four groups' cell parameters. (a)** Cell volume, **(b)** cell height and FWHM, and **(c)** mean height of particles of surface nanostructure and Rq.

To quantify the morphological and nanostructural differences between normal T lymphocytes and AM-treated T lymphocytes with different treatment times, a statistical analysis was performed as shown in Figure 5, including the changes of cell volume, cell height, FWHM, Rq, and particle mean height of surface nanostructure. These results demonstrated that the cell volume nearly double increased from 45 ± 5 μm^3^ (normal) to 84 ± 10 μm^3^ (10 μg/ml AM treatment for 48 h) (Figure 5a), which was in accordance with the increases of cell height (from 0.8 ± 0.1 μm to 1.5 ± 0.2 μm) and FWHM (from 5.5 ± 0.5 μm to 7 ± 1 μm) (Figure 5b). Interestingly, with the treatment time increasing to 72 h, the cell volume decreased to 33 ± 5 μm^3^ (10 μg/ml AM treatment for 72 h) (Figure 5a), which was also in accordance with the decreases of cell height (from 1.5 ± 0.2 μm to 0.4 ± 0.1 μm) and FWHM (from 7 ± 1 μm to 4 ± 0.5 μm) (Figure 5b). Following treatment with 10 μg/ml AM for 96 h, the cell volume was only about 1 μm^3^ and the height decreased to 150 nm (Figure 5a,b). When the measurements were conducted on nanoscale images, the results indicated that both Rq and the mean height of surface particles increased after being treated with AM for 48 and 72 h, which were positively correlated with reaction time (Figure 5c). But when the treatment time increased to 96 h, Rq and the mean height of surface particles decreased rapidly to about 60 ± 9 nm and 90 ± 9 nm (Figure 5c), which indicated that cell fragments became smaller.

These above AFM observation indicated that cell morphology changed significantly after treatment with AM for 48 h (Figure 2), for example, the cell height and FWHM increased obviously, which resulted in cell volume nearly double increasing. This result indicated that cells were swelling following treatment with AM for 48 h. In addition, the nanostructure images show that the cell membrane was damaged with several cracks and holes. More importantly than all of that, some debris was found on the cell surface and the substrate. These observed changes are in accord with the characteristics of oncosis described in the literature [13,32]. Further increase in reaction time (time = 72 h, AM concentration = 10 μg/ml) resulted in significant decreases of cell volume, cell height, and FWHM (Figure 3), and the cell nanostructure had been substantially changed, with surface particle diameter and Rq showing a particularly evident increase (Figure 5). Moreover, some larger and thicker fragments around the cell were found compared with T lymphocyte treatment for 48 h. These results demonstrated that T lymphocytes have lysed with increasing duration of exposure for 72 h, and the cell membrane was destroyed, which cause the leakage of cytoplasm and the decrease of cell volume. Furthermore, with increasing treatment time to 96 h, T lymphocytes were completely lysed and they existed in the form of smaller fragments. These smaller cell fragments could be easily cleared by phagocytes. These AFM images and the biophysical parameters of T lymphocyte visually demonstrated that AM may induce T lymphocyte oncosis.

### Changes in nanomechanical properties of the T lymphocyte with AM treatment

**Figure 6:**
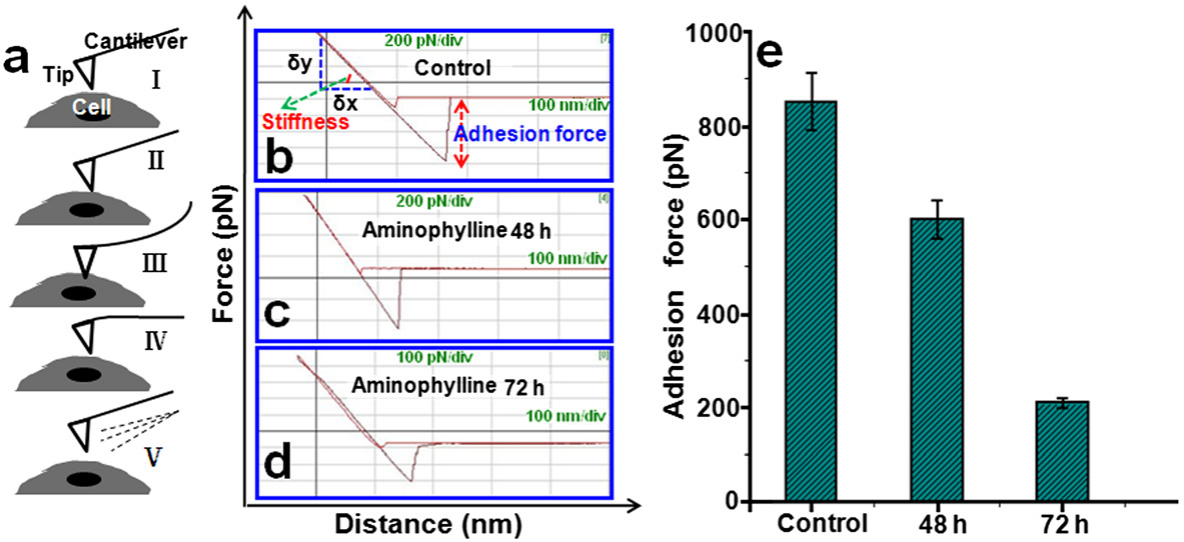
**Qualitative and quantitative analysis of nanomechanical properties of normal and AM-treated T lymphocytes, respectively. (a)** The principle diagram of force-distance curves and the approach (I-III) and retraction (IV-V) cycle. Three typical force-distance curves were recorded from normal T lymphocytes **(b)** and AM-treated T lymphocytes for 48 h **(c)** and 72 h **(d)**, respectively. **(e)** Histograms of statistical results of surface adhesion force, showing the adhesion force of T lymphocytes decreased gradually with increasing AM treatment time.

Previous studies have revealed that changes of cellular morphology may also lead to changes of cellular mechanical properties in some cases [10,24]. The interaction between medicaments and cells can induce changes in nanomechanical properties of cells [33]. While the mechanical properties of cancer cells and stem cells have been largely investigated [34,35], only a limited number of studies have focused on lymphocytes [10], and no study about the changes of nanomechanical properties of T lymphocytes treated with theophylline drugs was found. AFM is not only a surface imaging technique but also a sensitive force detector. Following the above morphology observation, in order to further prove that AM may induce T lymphocyte oncosis to exert anti-inflammatory effects for treating asthma, we therefore moved to study the mechanical properties of the T lymphocyte without and with 10 μg/ml AM treatment for 48 and 72 h (Figure 6).

Here, we used AFM-based force spectroscopy to extract adhesion force and stiffness of control and AM treatment groups, respectively. The operational principle of AFM force spectroscopy is schematically shown in Figure 6a. During tip approach (I, II, III), firstly, the tip was moved toward the cell surface (I) and (II) and then started to press samples at a constant loading force, which resulted in the deformation of both cantilever and soft samples (III). We can qualitatively analyze the stiffness of samples through the slope of approach curves *δy*/*δz* (Figure 6b). During tip retraction (IV, V), in the first place, the AFM tip began to retract and the deformation of cantilever began to recover (IV), and then the AFM tip broke away from the sample surface (V). When the tip-sample contact was ruptured, the adhesion force between the tip and samples was shown in the fore-distance curves (Figure 6b). Three representative force-distance curves recorded between the silicon nitride probe and the surface of the three groups' cells are shown in Figure 6b,c,d. The cell stiffness increased firstly and then decreased according to the qualitative analysis of the approaching branch of force-distance curves, but the cells treated with AM for 48 h were stiffer than both the normal cells and the cells treated with AM for 72 h. Stiffness of cells is a parameter of increasing importance in cellular physiology. On the one hand, cells mainly rely on cytoskeleton and osmotic pressure to maintain the basic morphology and function [36-39]. On the other hand, a filamentous cytoskeleton and osmotic pressure collectively govern the physical shape and mechanical properties (viscoelastic properties and stiffness) of eukaryotic cells [40-42]. Therefore, the increase of stiffness (48 h group) may be caused by the cytoskeleton reorganization and the generation of swelling pressure. When the AM treatment time increased to 72 h, the cells began to lyse, cellular pressure disappeared, and the cytoskeleton may be completely damaged, which resulted in the decrease of cell stiffness. Simultaneously, the adhesion force of the cell surface decreased to about two thirds of normal T lymphocytes after treatment with AM for 48 h (Figure 6e). Furthermore, with increasing duration of exposure (72 h), the adhesion force decreased more significantly (Figure 6e), which decreased to a quarter of the normal T lymphocytes' adhesion force. The composition of the cell membrane is mainly of phospholipid bilayer, carbohydrates, and proteins, which provide the cell surface with certain adhesion force. Cell adhesion is an essential prerequisite for survival, communication, and navigation of cells in organisms [43]. The decrease of cellular adhesion force suggested that the cell membrane was damaged, which was in accord with the results of AFM images (Figures 2, 3, and 4). These qualitative and quantitative data of cells' nanomechanical properties further illustrates that AM could induce T lymphocyte oncosis.

## Conclusions

Changes in the morphological and mechanical properties of T lymphocytes induced by AM were first studied by AFM at the nanoscale and piconewton level. By treatment with 10 μg/ml AM for 48, 72, and 96 h, T lymphocytes' biophysical parameters including cell volume, cell height, FWHM, mean height of particles, Rq, the adhesion force, and the stiffness all changed regularly. With increasing AM treatment time, T lymphocytes' volume nearly double increased and then decreased, while membrane roughness showed a particularly evident increase. Analysis of force-distance curves showed that the cell stiffness increased firstly and then decreased with the AM treatment time increasing from 48 to 72 h, while the adhesion force decreased from two thirds to a quarter of normal T lymphocytes' adhesion force, respectively. These changes in cell morphology, surface nanostructures, and nanomechanical properties were closely correlated to cell swelling, oncosis, and lysis. We established a visual method by analyzing the changes of biophysical parameters of cells to reveal the characteristics and process of oncosis. Combined with clinical research and our present data, our nano-spatial findings provide new insights into the mechanism of AM curing asthma: (i) T lymphocyte oncosis should be induced, and cell fragments in the airway of the asthmatic should be generated. (ii) Phagocytes clear up debris and further clear all the T lymphocyte that was blocking the airway of the asthmatic.

## Abbreviations

AFM: atomic force spectroscopy; AM: aminophylline; FWHM: full width at half maximum; Rq: root-mean-square roughness.

## Competing interests

The authors declare that they have no competing interests.

## Authors' contributions

XH carried out the experiment, fabrication of samples, and AFM measurements and drafted the manuscript. JH carried out the AFM analysis and statistical analysis and participated in the drafting of the manuscript. ML revised the manuscript. CZ initiated, planned, controlled the research process, and revised the manuscript. All authors read and approved the final manuscript.

## References

[B1] Mylonaki E Manika K Zarogoulidis P Domvri K Voutsas V Zarogoulidis K Mourelatos D In vivo synergistic cytogenetic effects of aminophylline on lymphocyte cultures from patients with lung cancer undergoing chemotherapy Mutat Res 2012 740 1 5 10.1016/j.mrfmmm.2012.10.002 23116732

[B2] Shiohira H Fujii M Koizumi N Kondoh M Watanabe Y Novel chronotherapeutic rectal aminophylline delivery system for therapy of asthma Int J Pharm 2009 379 119 124 10.1016/j.ijpharm.2009.06.017 19555748

[B3] Prigal SJ Fuchs AM Schulman PM The treatment of asthma with rectal suppositories of aminophylline and sodium pentobarbital J Allergy 1946 17 172 177 10.1016/0021-8707(46)90008-1 21027126

[B4] D'Avila RS Piva JP Cauduro Marostica PJ Amantea SL Early administration of two intravenous bolus of aminophylline added to the standard treatment of children with acute asthma Respir Med 2008 102 156 161 10.1016/j.rmed.2007.07.030 17869497

[B5] Billington CK Ojo OO Penn RB Ito S cAMP regulation of airway smooth muscle function Pulm Pharmacol Ther 2013 26 112 120 10.1016/j.pupt.2012.05.007 22634112 PMC3574867

[B6] Kizawa Y Furuya M Saito K Masuko T Kusama T Effects of dexamethasone and aminophylline on survival of Jurkat and HL-60 cells Biol Pharm Bull 2006 29 281 285 10.1248/bpb.29.281 16462032

[B7] Szczypka M Obminska-Mrukowicz B Modulating effects of nonselective and selective phosphodiesterase inhibitors on lymphocyte subsets and humoral immune response in mice Pharmacol Rep 2010 62 1148 1158 10.1016/S1734-1140(10)70377-7 21273672

[B8] Lin CC Lin CY Liaw SF Chen A Pulmonary function changes and immunomodulation of Th 2 cytokine expression induced by aminophylline after sensitization and allergen challenge in brown Norway rats Ann Allergy Asthma Immunol 2002 88 215 222 10.1016/S1081-1206(10)61999-0 11868928

[B9] Costa DL Guimaraes LH Cardoso TM Queiroz A Lago E Roselino AM Bacellar O Carvalho EM Silva JS Characterization of regulatory T cell (Treg) function in patients infected with *Leishmania braziliensis* Hum Immunol 2013 74 1491 1500 10.1016/j.humimm.2013.08.269 23993989 PMC3846617

[B10] Hu M Wang J Zhao H Dong S Cai J Nanostructure and nanomechanics analysis of lymphocyte using AFM: from resting, activated to apoptosis J Biomech 2009 42 1513 1519 10.1016/j.jbiomech.2009.03.051 19477449

[B11] Maslan J Mims JW What is asthma? Pathophysiology, demographics, and health care costs Otolaryngol Clin North Am 2014 47 13 22 10.1016/j.otc.2013.09.010 24286675

[B12] Renauld JC New insights into the role of cytokines in asthma J Clin Pathol 2001 54 577 589 10.1136/jcp.54.8.577 11477111 PMC1731485

[B13] Zhou X Konkel ME Call DR Type III secretion system 1 of *Vibrio parahaemolyticus* induces oncosis in both epithelial and monocytic cell lines Microbiology 2009 155 837 851 10.1099/mic.0.024919-0 19246755

[B14] Hein S Arnon E Kostin S Schonburg M Elsasser A Polyakova V Bauer EP Klovekorn WP Schaper J Progression from compensated hypertrophy to failure in the pressure-overloaded human heart: structural deterioration and compensatory mechanisms Circulation 2003 107 984 991 10.1161/01.CIR.0000051865.66123.B7 12600911

[B15] Lekka M Laidler P Applicability of AFM in cancer detection Nat Nanotechnol 2009 4 72 10.1038/nnano.2009.036 19197298

[B16] Stolz M Gottardi R Raiteri R Miot S Martin I Imer R Staufer U Raducanu A Dueggelin M Baschong W Daniels AU Friederich NF Aszodi A Aebi U Early detection of aging cartilage and osteoarthritis in mice and patient samples using atomic force microscopy Nat Nanotechnol 2009 4 186 192 10.1038/nnano.2008.410 19265849

[B17] Titushkin I Cho M Modulation of cellular mechanics during osteogenic differentiation of human mesenchymal stem cells Biophys J 2007 93 3693 3702 10.1529/biophysj.107.107797 17675345 PMC2072058

[B18] Bozna BL Polzella P Rankl C Zhu R Salio M Shepherd D Duman M Cerundolo V Binding strength and dynamics of invariant natural killer cell T cell receptor/CD1d-glycosphingolipid interaction on living cells by single molecule force spectroscopy J Biol Chem 2011 286 15973 15979 10.1074/jbc.M110.192674 21454514 PMC3091206

[B19] Shi Q Luo S Jia H Feng L Lu X Zhou L Cai J Insulin-producing cells could not mimic the physiological regulation of insulin secretion performed by pancreatic beta cells Nanoscale Res Lett 2013 8 1 8 10.1186/1556-276X-8-1 23421382 PMC3585706

[B20] El-Kirat-Chatel S Dufrene YF Nanoscale imaging of the Candida - macrophage interaction using correlated fluorescence-atomic force microscopy ACS Nano 2012 6 10792 10799 23146149 10.1021/nn304116f

[B21] Dupres V Menozzi FD Locht C Clare BH Abbott NL Cuenot S Bompard C Raze D Dufrene YF Nanoscale mapping and functional analysis of individual adhesins on living bacteria Nat Methods 2005 2 515 520 10.1038/nmeth769 15973422

[B22] Huang X Li X Wang Q Dai J Hou J Chen L Single-molecule level binding force between collagen and collagen binding domain-growth factor conjugates Biomaterials 2013 34 6139 6146 10.1016/j.biomaterials.2013.04.057 23706541

[B23] Hosseini BH Louban I Djandji D Wabnitz GH Deeg J Bulbuc N Samstag Y Gunzer M Spatz JP Haemmerling GJ Immune synapse formation determines interaction forces between T cells and antigen-presenting cells measured by atomic force microscopy Proc Natl Acad Sci 2009 106 17852 17857 10.1073/pnas.0905384106 19822763 PMC2764924

[B24] Pi J Yang F Jin H Huang X Liu R Yang P Cai J Selenium nanoparticles induced membrane bio-mechanical property changes in MCF-7 cells by disturbing membrane molecules and F-actin Bioorg Med Chem Lett 2013 23 6296 6303 10.1016/j.bmcl.2013.09.078 24140445

[B25] Vadillo-Rodriguez V Beveridge TJ Dutcher JR Surface viscoelasticity of individual gram-negative bacterial cells measured using atomic force microscopy J Bacteriol 2008 190 4225 4232 10.1128/JB.00132-08 18408030 PMC2446760

[B26] Cross SE Jin Y-S Rao J Gimzewski JK Nanomechanical analysis of cells from cancer patients Nat Nanotechnol 2007 2 780 783 10.1038/nnano.2007.388 18654431

[B27] Das SK Das AR Guha AK Structural and nanomechanical properties of *Termitomyces clypeatus* cell wall and its interaction with chromium(VI) J Phys Chem B 2009 113 1485 1492 10.1021/jp808760f 19146378

[B28] Xing F Wang J Hu M Yu Y Chen G Liu J Comparison of immature and mature bone marrow-derived dendritic cells by atomic force microscopy Nanoscale Res Lett 2011 6 1 9 10.1186/1556-276X-6-455 PMC3211875 21762525

[B29] Wang J Wan Z Liu W Li L Ren L Wang X Sun P Ren L Zhao H Tu Q Zhang Z Song N Zhang L Atomic force microscope study of tumor cell membranes following treatment with anti-cancer drugs Biosens Bioelectron 2009 25 721 727 10.1016/j.bios.2009.08.011 19734031

[B30] Sato K Adachi T Ueda D Hojo M Tomita Y Measurement of local strain on cell membrane at initiation point of calcium signaling response to applied mechanical stimulus in osteoblastic cells J Biomech 2007 40 1246 1255 10.1016/j.jbiomech.2006.05.028 16887125

[B31] Schempp CM Kirkin V Simon-Haarhaus B Kersten A Kiss J Termeer CC Gilb B Kaufmann T Borner C Sleeman JP Simon JC Inhibition of tumour cell growth by hyperforin, a novel anticancer drug from St. John's wort that acts by induction of apoptosis Oncogene 2002 21 1242 1250 10.1038/sj.onc.1205190 11850844

[B32] Majno G Joris I Apoptosis, oncosis, and necrosis: an overview of cell death Am J Pathol 1995 146 3 15 7856735 PMC1870771

[B33] Lam WA Rosenbluth MJ Fletcher DA Chemotherapy exposure increases leukemia cell stiffness Blood 2007 109 3505 3508 10.1182/blood-2006-08-043570 17179225 PMC1852256

[B34] Gonzalez-Cruz RD Fonseca VC Darling EM Cellular mechanical properties reflect the differentiation potential of adipose-derived mesenchymal stem cells Proc Natl Acad Sci 2012 109 1523 1529 10.1073/pnas.1120349109 PMC3386052 22615348

[B35] Thomas G Burnham NA Camesano TA Wen Q Measuring the mechanical properties of living cells using atomic force microscopy J Vis Exp 2013 doi:10.3791/50497 10.3791/50497 PMC3729185 23851674

[B36] Sims JR Karp S Ingber DE Altering the cellular mechanical force balance results in integrated changes in cell, cytoskeletal and nuclear shape J Cell Sci 1992 103 1215 1222 1487498 10.1242/jcs.103.4.1215

[B37] Liass LA Vasil'ev IM Changes in the morphology and cytoskeleton of cultured fibroblasts as a result of raised osmotic pressure Biull Eksp Biol Med 1981 92 93 96 7197563

[B38] Drake T Vavylonis D Model of fission yeast cell shape driven by membrane-bound growth factors and the cytoskeleton PLoS Comput Biol 2013 9 1003287 10.1371/journal.pcbi.1003287 PMC3798282 24146607

[B39] Maiworm AI Presta GA Santos-Filho SD De Paoli S Giani TS Fonseca AS Bernardo-Filho M Osmotic and morphological effects on red blood cell membrane: action of an aqueous extract of *Lantana camara* Revista Brasileira De Farmacognosia-Braz J Pharmacognosy 2008 18 42 46

[B40] Wang S Arellano-Santoyo H Combs PA Shaevitz JW Actin-like cytoskeleton filaments contribute to cell mechanics in bacteria Proc Natl Acad Sci 2010 107 9182 9185 10.1073/pnas.0911517107 20439764 PMC2889055

[B41] Guilak F Erickson GR Ting-Beall HP The effects of osmotic stress on the viscoelastic and physical properties of articular chondrocytes Biophys J 2002 82 720 727 10.1016/S0006-3495(02)75434-9 11806914 PMC1301881

[B42] Zhou EH Trepat X Park CY Lenormand G Oliver MN Mijailovich SM Hardin C Weitz DA Butler JP Fredberg JJ Universal behavior of the osmotically compressed cell and its analogy to the colloidal glass transition Proc Natl Acad Sci 2009 106 10632 10637 10.1073/pnas.0901462106 19520830 PMC2695406

[B43] Benoit M Selhuber-Unkel C Measuring cell adhesion forces: theory and principles Atomic Force Microscopy in Biomedical Research: Methods and Protocols 2011 Berlin: Springer 355 377 10.1007/978-1-61779-105-5_21 21660737

